# Extended X-ray absorption fine structure spectroscopy measurements and *ab initio* molecular dynamics simulations reveal the hydration structure of the radium(II) ion

**DOI:** 10.1016/j.isci.2022.104763

**Published:** 2022-07-19

**Authors:** Akiko Yamaguchi, Kojiro Nagata, Keita Kobayashi, Kazuya Tanaka, Tohru Kobayashi, Hajime Tanida, Kojiro Shimojo, Tetsuhiro Sekiguchi, Yui Kaneta, Shohei Matsuda, Keiichi Yokoyama, Tsuyoshi Yaita, Takashi Yoshimura, Masahiko Okumura, Yoshio Takahashi

**Affiliations:** 1 Center for Computational Science and e-Systems, Japan Atomic Energy Agency, 148-4 Kashiwanoha Campus, 178-4 Wakashiba, Kashiwa, Chiba 277-0871, Japan; 2 Advanced Science Research Center, Japan Atomic Energy Agency, 2-4 Shirakata, Tokai-mura, Naka-gun, Ibaraki 319-1195, Japan; 3 Department of Earth and Planetary Science, Graduate School of Science, The University of Tokyo, 7-3-1 Hongo, Bunkyo, Tokyo 113-0033, Japan; 4 Materials Sciences Research Center, Japan Atomic Energy Agency, 2-4 Shirataka, Tokai-mura, Naka-gun, Ibaraki 319-1195, Japan; 5 Radioisotope Research Center, Institute for Radiation Sciences, Osaka University, 2-4 Yamadaoka, Suita, Osaka 565-0871, Japan

**Keywords:** Nuclear structure, Nuclear spectroscopy, Molecular dynamics

## Abstract

Radium is refocused from the viewpoint of an environmental pollutant and cancer therapy using alpha particles, where it mainly exists as a hydrated ion. We investigated the radium hydration structure and the dynamics of water molecules by extended X-ray absorption fine structure (EXAFS) spectroscopy and *ab initio* molecular dynamics (AIMD) simulation. The EXAFS experiment showed that the coordination number and average distance between radium ion and the oxygen atoms in the first hydration shell are 9.2 ± 1.9 and 2.87 ± 0.06 Å, respectively. They are consistent with those obtained from the AIMD simulations, 8.4 and 2.88 Å. The AIMD simulations also revealed that the water molecules in the first hydration shell of radium are less structured and more mobile than those of barium, which is an analogous element of radium. Our results indicate that radium can be more labile than barium in terms of interactions with water.

## Highlights

• Extended X-ray absorption fine structure (EXAFS) measurement revealed the hydration structure of radium ion
• *Ab initio* molecular dynamics (AIMD) simulation brought consistent results
• AIMD revealed the structural and dynamic properties of the water molecules
• The hydration structure of radium ion is more labile than that of barium ion

## Graphical abstract

**Figure:**
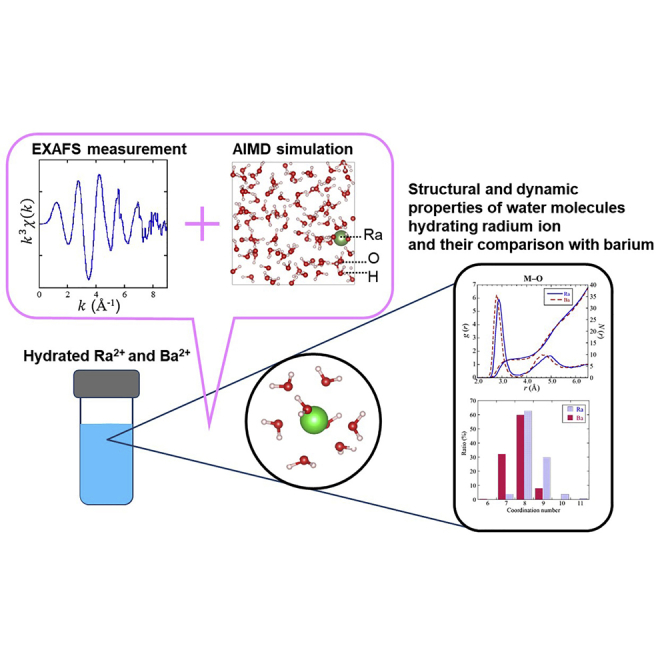

## Introduction

Radium (Ra) is a popular radioactive element, having no stable isotopes and adverse health effects. Ra has not been in the focus of environmental science for a long time owing to its rarity. Since the 1980s, the concentration of Ra in drinking and groundwater gained the attention of the scientific community (Hess et al., 1985; Zhuo et al., 2001; Szabo et al., 2012; Guo et al., 2018). In addition, the environmental transport of Ra is crucial for geological disposal of high-level radioactive waste, such as spent fuel because ^226^Ra is a descendant nuclide of ^238^U, which has a long half-life (1,600 years). The concentration of Ra in the environment depends on various factors, such as pH, redox state, ionic strength, and mineral component. The amount of Ra tends to be higher in an anoxic environment (Szabo et al., 2012), causing a higher concentration in deeper groundwater than in surface water. Taking advantage of this trend, Ra can be used as a natural tracer for detecting submarine groundwater discharge (Burnett et al., 2006). Ra is also used for dating volcanic ejecta (Tanguy et al., 2007) and buried sediment (Olley et al., 1996), using the uranium and thorium decay chains. However, shale gas extraction using new drilling techniques, such as horizontal drilling and hydraulic fracturing, has become active recently, raising concerns about the contamination of Ra in surface and drinking water (Vengosh et al., 2014). As described above, the long-lived isotope of Ra such as ^226^Ra is basically toxic. However, ^223^Ra with a half-life of 11.43 days has recently been used to treat bone metastasis in castration-resistant prostate cancer (CRPC) (Deshayes et al., 2017; Delgado Bolton and Giammarile, 2018; Morris et al., 2019).

Ra mainly exists as a hydrated divalent cation in the environment and does not form any pure Ra minerals because of its low concentration (Langmuir et al., 1985). Therefore, the environmental behavior of Ra^2+^ is governed by coprecipitation, recrystallization, and adsorption reactions with other alkaline earth elements (Kraemer and Reid, 1984; Langmuir and Melchior, 1985; Beaucaire and Toulhoat, 1987; Vinson et al., 2013). The adsorption of Ra^2+^ on clay minerals and iron (hydr)oxides has also been reported (Martin et al., 2003; Hidaka et al., 2007). This behavior has also been studied by numerous laboratory experiments (Doerner and Hoskins, 1925; Curti et al., 2010; Hedstrom et al., 2013; Chen and Kocar, 2018). For example, the early uptake of Ra^2+^ during the recrystallization and coprecipitation of barite (barium sulfate), has been observed (Hedstrom et al., 2013). Similar chemical reactions were applied to bone metastasis in CRPC in the field of nuclear medicine, substituting ^223^Ra for calcium in hydroxyapatite complexes. Atomic-level investigation of hydration and dehydration of Ra^2+^ is essential to understand these reactions such as adsorption and coprecipitation which occur at the solid-water interface with hydration and/or dehydration processes. However, the hydration properties of Ra^2+^ have not been well studied.

Numerical simulations are effective in studying the hydration structure of Ra^2+^ safely. The formation of Ra^2+^ hydrated clusters was studied via static quantum mechanical calculations. Namely, the fragment molecular orbital–molecular dynamics simulation approach was applied to study the hydration structures in an aqueous solution (Matsuda and Mori, 2014b). Classical molecular dynamics, *ab initio* quantum chemical calculations with the relativistic model core potential (MCP) method, and fragment molecular orbital–molecular dynamics were conducted to study the hydration of Ra^2+^ and compare it with other hydrated divalent alkaline earth metal ions (Matsuda and Mori, 2014a). Recently, classical molecular dynamics simulation with the force field fitted to the results of quantum mechanical calculations was conducted (Pappalardo et al., 2021). In the hydrated barium ion (Ba^2+^), an analog element of Ra cases, it has been shown that full-*ab initio* molecular dynamics (AIMD) simulation is one of the best simulation methods compared with other methods, including QM/MM, a hybrid method of quantum and classical calculations, in the accuracy for reproducing experimental results (Yamaguchi et al., 2021). The full-AIMD simulations have not been applied to hydrated Ra^2+^ systems mainly owing to the higher computational cost of AIMD than those of other methods. However, the recent development of supercomputers enables large-scale full-AIMD simulations even for large systems such as the hydrated Ra^2+^ systems with a large number of water molecules.

There are some experimental methods to directly investigate the hydration structure at the atomic level, such as extended X-ray absorption fine structure (EXAFS) measurement, X-ray and neutron diffraction measurements, vibration spectroscopic measurement, and nuclear magnetic resonance (Marcus, 2010). EXAFS is suitable for studying the hydration structures of Ra^2+^ owing to its high selectivity of elements and high sensitivity, which are crucial for measuring a dilute solution such as hydrated Ra^2+^. As mentioned above, no spectroscopic experiment has been performed on hydrated Ra^2+^ because of the radiation toxicity of Ra. However, some facilities have beamlines to measure radioactive materials. SPring–8, a synchrotron radiation facility in Japan, has two beamlines that can be applied to radioactive materials.

Therefore, in this study, EXAFS was applied for the first time to determine the hydration structure of Ra^2+^ using the BL22XU beamline of SPring–8. Additionally, the AIMD simulations in this study were conducted to evaluate the average hydration structure and the dynamic properties of the hydration structure. These methods are complementary, and their combination revealed the microscopic hydration structure of Ra^2+^ and its dynamics. Our study clarifies the differences between the hydration properties of Ra^2+^ and Ba^2+^, which is often used as a non-radioactive analog of Ra^2+^.

## Results and discussion

### Hydration structure in comparison between extended X-ray absorption fine structure and *ab initio* molecular dynamics results

**Figure 1:**
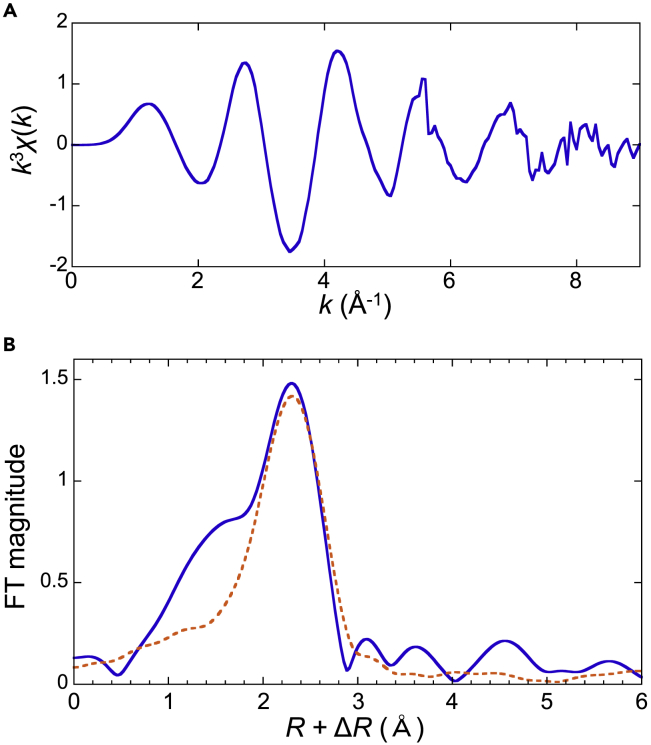
EXAFS spectrum EXAFS spectrum of hydrated Ra^2+^ in (A) k-space and (B) R-space. The horizontal and vertical axes represent k (Å^−1^) and $k^{3}\chi \left(k\right)$ in (A) and interatomic distance and the Fourier transformation magnitude in (B), where χ(k) is EXAFS oscillation. The dotted line represents the fitted result.

The EXAFS analysis showed that the average distance between the Ra^2+^ and the oxygen atoms of the water molecules in the first hydration shell (${\bar{r}}_{Ra–O}$) and coordination number (CN) were 2.87 ± 0.06 Å and 9.2 ± 1.9, respectively (Figure 1 and Table 1). As this is the first atomic-level structural information obtained for hydrated Ra^2+^, these values could not be validated by direct comparison with other spectroscopic methods. Hence, we considered the consistency with the experimental results of other alkaline earth metal ions. Table 2 shows the monotonic increase in the distance between the cation and oxygen atoms in the hydration water molecules, obtained using various methods, including EXAFS, with the atomic number. The effective ionic radii shown in previous studies (Shannon, 1976) have the same tendency (Table 2), and the value of Ra^2+^ is consistent with the trend. We also evaluated the effective radius of a water molecule, defined by the difference between the interatomic distance and the effective ionic radius (Marcus, 1988). The radius of the water molecule hydrated to Ra^2+^ was determined to be 1.39 Å. This value is almost the same as other alkaline earth metal elements (Table 2) and as the radius of O^2−^ used for calculating the effective ionic radius (1.40 Å) (Shannon, 1976). These results mean that the interatomic distance between Ra^2+^ and oxygen atoms determined by the experimental EXAFS method in this study is consistent with the results of other alkaline earth metal elements, confirming the adequacy of the obtained values. The CN is directly related to the cation–oxygen distance because a longer cation–oxygen distance implies a larger shell volume for water molecules. Therefore, the observed CN is consistent with other alkaline earth metal elements (Table 2).

**Table 1:** Extended X-ray absorption fine structure spectroscopy parameters for fitting

k range (Å^−1^)	Shell	CN	${\bar{r}}_{Ra–O}$ (Å)	$\text{Δ}E_{0}$ (eV)	$\sigma^{2}$ (× 10^−3^ Å^2^)	$C_{3}$ (× 10^−4^ Å^3^)	$R_{f}$ (%)
2.3–9.0	Ra–O	9.2 ± 1.9	2.87 ± 0.06	6.3 ± 2.1	26.5 ± 0.9	7.3 ± 38.2	1.29

CN, ${\bar{r}}_{Ra–O}$, $\text{Δ}E_{0}$, $\sigma^{2}$, $C_{3}$, and $R_{f}$ represent the coordination number, the averaged interatomic distance between Ra^2+^ and nearest oxygen atoms of water molecules, threshold $E_{0}$ shift, DW factor, the third-order cumulant, and residual factor.

**Table 2:** The averaged distance between ion (M) and the oxygen atoms of water molecules in the first hydration shell (${\bar{r}}_{M–O}$) and coordination number (CN) observed via experiments, effective ionic radii (EIR) and CN, and the differences between ${\bar{r}}_{M–O}$ and EIR for alkali earth metal ions

Ion (M)	${\bar{r}}_{M–O}$ (Å)	CN	EIR (Å) (Shannon, 1976)	CN (Shannon, 1976)	${\bar{r}}_{M–O}-EIR$ (Å)	Method
Ra^2+^	2.87 ± 0.06 (This study)	9.2 ± 1.9 (This study)	1.48	8	1.39	EXAFS
Ba^2+^	2.79 ± 0.02 (Yamaguchi et al., 2021)	8.0 ± 1.9 (Yamaguchi et al., 2021)	1.42	8	1.37	EXAFS
Sr^2+^	2.64 ± 0.01 (Yokoyama, 1995)	8.1 ± 0.1 (Yokoyama, 1995)	1.26	8	1.38	XRD and ND
Ca^2+^	2.40 ± 0.04 (Yokoyama, 1995)	6.3 ± 0.7 (Yokoyama, 1995)	1.00	6	1.40	XRD and ND
Mg^2+^	2.09 ± 0.04 (Yokoyama, 1995)	6.0 ± 0.1 (Yokoyama, 1995)	0.72	6	1.37	XRD

XRD and ND means X-ray diffraction and neutron diffraction, respectively. The values of Sr^2+^, Ca^2+^, and Mg^2+^ reported in the literature (Yokoyama, 1995) were average of the reported values in literature (Ohtaki and Radnai, 1993; Persson et al., 1995).

AIMD simulation is a reliable method for comparison with experimental results. In the Ba^2+^ case, the results obtained by EXAFS and AIMD simulations are consistent (Yamaguchi et al., 2021). We note that the results of AIMD simulations depend on the exchange-correlation functionals and the strongly constrained and appropriately normed (SCAN) XC functional showed good agreement with EXAFS experiment results. Therefore, the results using the SCAN XC functional are mainly discussed in this study. The Supplementary Information shows the comparison of the results with several other XC functionals (Figures S3–S8 and Table S1).

**Figure 2:**
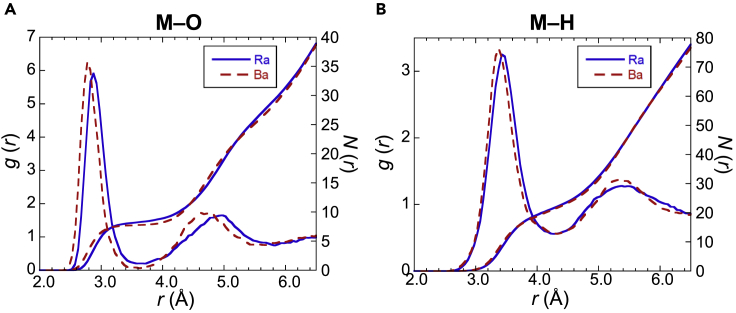
Radial distribution functions and running integration numbers Radial distribution functions g(r) and running integration numbers N(r) of (A) M−O and (B) M−H evaluated using the AIMD simulation; M represents the metal ion, Ba^2+^ or Ra^2+^.

The radial distribution functions (RDFs), g(r), of Ra–O and radium–hydrogen (Ra–H) were evaluated by AIMD simulations (Figure 2). The running integration numbers, N(r), of Ra–O and Ra–H were obtained by the RDFs. The ${\bar{r}}_{Ra–O}$ and CN can be obtained from the g(r) and N(r), and compared with the experimental EXAFS results. The g(r) of Ra–O has two peaks corresponding to the first and second hydration shells (Figure 2A). The first peak position, i.e., 2.88 Å of g(r) for Ra–O, is consistent with ${\bar{r}}_{\text{Ra}–\text{O}}$ as determined by the EXAFS experiment (2.87 ± 0.06 Å). This result is consistent with the results predicted by other simulation methods (Table 3). The CN is defined by the value of N(r) at the first minimum of g(r), i.e., 8.4. This value is consistent with the experimental value (9.2 ± 1.9). All of the ${\bar{r}}_{\text{Ra}–\text{O}}$ and CN values obtained with several XC functionals are consistent with the experimental results (Figure S3).

**Table 3:** Comparison of hydration structures of Ba^2+^ and Ra^2+^. ${\bar{r}}_{M–O}$, CN, tilt angle (Ψ), Debye–Waller factor ($\sigma^{2}$), and ε for Ra^2+^ and Ba^2+^

M		Method	${\bar{r}}_{M–O}$ (Å)	CN	Ψ(°)	$\sigma^{2}$ (Å^2^)	ε (Å)	Ref.
Ra^2+^	Exp.	EXAFS	2.87 ± 0.06	9.2 ± 1.9	–	0.027	–	This study
Ra^2+^	Sim.	AIMD SCAN	2.88	8.4	132	0.041	0.31	This study
Ra^2+^	Sim.	FMO-MD	2.85	8.1	–	–	–	Previous study (Matsuda and Mori, 2014b)
Ra^2+^	Sim.	MCP	2.80–2.95	7–9	–	–	–	Previous study (Matsuda and Mori, 2014a)
Ra^2+^	Sim.	MD	2.93	9.8	135	0.034	0.24	Previous study (Pappalardo et al., 2021)
Ba^2+^	Exp.	EXAFS	2.79 ± 0.02	8.0 ± 1.9	–	0.020	–	Previous study (Yamaguchi et al., 2021)
Ba^2+^	Exp.	EXAFS	2.81 ± 0.03	8.1 ± 0.3	–	–	–	Previous study (Persson et al., 1995)
Ba^2+^	Exp.	EXAFS	2.85 ± 0.02	8.0 ± 0.2	–	–	–	Previous study (Migliorati, Caruso and D’Angelo, 2019)
Ba^2+^	Sim.	AIMD SCAN	2.78	7.8	138	0.031	0.26	This study and previous study (Yamaguchi et al., 2021)
Ba^2+^	Sim.	AIMD PW91	2.80	8.0	–	–	–	Previous study (Chaudhari et al., 2015)
Ba^2+^	Sim.	MD	2.81	9.4	138	0.034	0.22	Previous study (Pappalardo et al., 2021)
Ba^2+^	Sim.	MD	2.85	8.1	–	–	–	Previous study (Migliorati, Caruso and D’Angelo, 2019)
Ba^2+^	Sim.	QM/MM	2.86	9.3	–	–	–	Previous study (Hofer et al., 2005)

The RDF of Ra–H also has two peaks corresponding to the first and second shells (Figure 2B). The value of g(r) for Ra–H at the first minimum (0.6) is much larger than that of Ra–O (0.2). In addition, the CN of hydrogen is 21.5, which deviates from the number of protons expected by the CN of oxygen (8.4×2). This deviation can be explained by the rotation of water molecules in the first and second shells, which increases the g(r) of Ra–H between the first and second shells. This trend is also shown in the results of Ba^2+^.

### Structural properties of water molecules around radium ion

**Figure 3:**
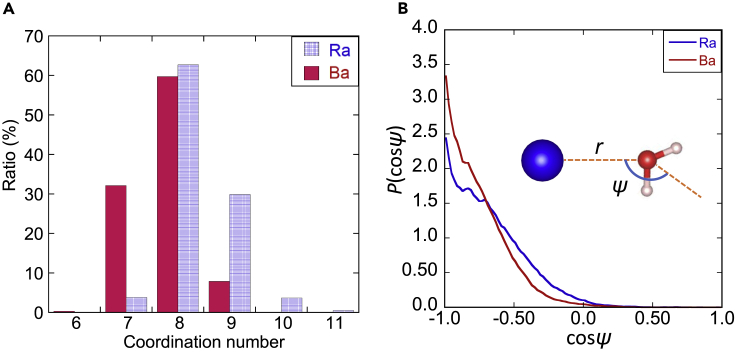
Histograms of coordination numbers and probability distribution of tilt angle (A) Histograms of coordination numbers and (B) the probability distribution of tilt angle (ψ) of water molecules hydrating of Ba^2+^ and Ra^2+^ evaluated using the AIMD simulation. As for Figure 3B, the horizontal and vertical axes represent the cosine of the tilt angle ψ and the probability distribution P(cosψ), respectively. The inset shows the simple model with a cation and a water molecule discussed in the text. The probability distribution of the tilt angle P(cosψ) is a monotonically decreasing function of the distance between the cation and oxygen in the water molecule r. See the text for details.

The AIMD simulation results were analyzed in more detail to obtain the structural properties of water molecules hydrating Ra^2+^ and Ba^2+^. The AIMD simulation showed that the histogram of the number of water molecules in the first hydration shell fluctuates (Figure 3A). In Ra^2+^, the eight- and nine-coordinate structures are primary and secondary, respectively, and the seven- and ten-coordinate structures appear rarely. The probability distribution for Ba^2+^ differs from Ra^2+^, i.e., the primary structure is the same, but the secondary and tertiary structures are seven- and nine-coordinate, respectively. The difference between the CNs of Ra^2+^ and Ba^2+^ reflects these differences between the ancillary structures. The larger secondary structure of Ra^2+^ can be explained by the difference in ionic radii of Ra^2+^ and Ba^2+^, i.e., a larger ionic radius provides a larger first hydration shell volume that can contain a greater number of water molecules.

The tilt angle between the dipole vector of a water molecule in the first shell and the vector pointing from the water oxygen to the metal ion is a good indicator of the structural property of the water molecule. Figure 3B shows the flatter probability distribution of the tilt angles P(cosψ) for Ra^2+^ than Ba^2+^. The flatter distribution implies that the collision between water molecules easily changes the direction of the dipole vector. To understand the difference, we considered a simple model that consists of a cation (M) and a water molecule shown in the inset of Figure 3B. Obviously, the energy to tilt the dipole vector is a monotonic decrease function of the ${\bar{r}}_{M–O}$. The ${\bar{r}}_{Ba–O}$ is shorter than the ${\bar{r}}_{Ra–O}$ (Table 3). Therefore, the direction of the dipole vector of a water molecule in the first shell of Ra^2+^ can be changed more readily than that of Ba^2+^. This explains the origin of the flatter distribution of P(cosψ) for Ra^2+^.

The AIMD simulations were also used to evaluate the second cumulants (SCs) of the $r_{Ra–O}$ and $r_{Ba–O}$ (Table 3). SC is defined as follows:

(Equation 1) $$\begin{array}{l} \sigma^{2}=\langle {\left(r_{M–O}-{\bar{r}}_{M–O}\right)}^{2}\rangle \end{array}$$

where $r_{M–O}$, ${\bar{r}}_{M–O}$, and X represent the distance between an ion M and the oxygen atoms of the water molecules in the first hydration shell, its average, and the average of X, respectively.

SC is associated with the Debye–Waller (DW) factor in spectroscopic experiments. The value of the SC for Ra^2+^ (0.041 Å^2^) is slightly larger than that for Ba^2+^ (0.031 Å^2^). Their relationship is consistent with the higher DW factor of Ra^2+^ in the EXAFS experiments (Table 3). Comparison between these results implies that the hydration structure of Ra^2+^ is more disordered and flexible than that of Ba^2+^. This tendency has been confirmed by the simulation results with other XC functionals.

Furthermore, the eccentricity ε was evaluated for Ra^2+^ and Ba^2+^ via AIMD simulations (Table 3). ε is the average distance between the cation and center of mass of the water molecules in the first hydration shell, defined in a previous study (Caralampio et al., 2017) as follows:

(Equation 2) $$\begin{array}{l} \varepsilon \equiv \langle r_{M–CMO}\rangle \end{array}$$

where CMO represents the center of mass of the water molecules in the first hydration shell. The obtained values were 0.31 Å (Ra^2+^) and 0.26 Å (Ba^2+^), which were compared with the classical MD results (Pappalardo et al., 2021) of 0.24 Å (Ra^2+^) and 0.22 Å (Ba^2+^). Consistent result of the ε value of Ra^2+^ being higher than that of Ba^2+^ was obtained via the simulation method. These results imply that the hydration shell of Ba^2+^ is structured to a greater extent than that of Ra^2+^.

### Dynamic properties of water molecules around radium ion

**Figure 4:**
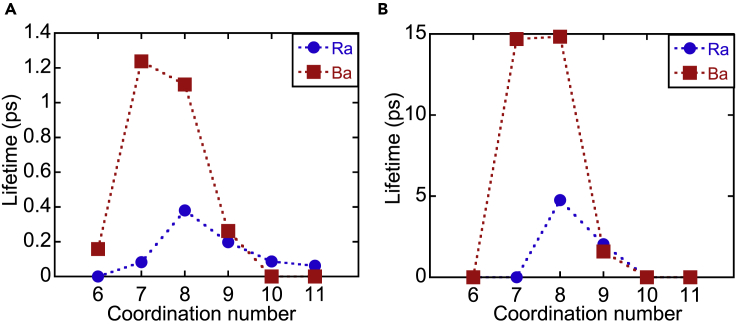
Lifetimes of the CN Lifetimes of the CN of Ba^2+^ and Ra^2+^ for (A) $t^{*}=0$ ps and (B) $t^{*}=0.5$. The horizontal and vertical axes represent CN and lifetime (ps), respectively. Red square and blue circle represent the lifetimes of the CN of Ba^2+^ and Ra^2+^, respectively.

The hydration structure is not static but dynamic. To evaluate the dynamical property, the mean residence time (MRT) of water molecules in the first hydration shell was evaluated by the “direct” method (Hofer et al., 2004), which is defined as

(Equation 3) $$\begin{array}{l} MRT\;\left(t^{*}\right)=\;\frac{t_{samp}CN_{av}}{N_{ex}^{t^{*}}} \end{array}$$

where $t_{samp}$, $CN_{av}$, and $N_{ex}^{t^{*}}$ are the sampling time (50 ps), average CN, and number of the exchange events that lasted longer $t^{*}$ ps, respectively. The MRTs for Ra^2+^ with $t^{*}=0$ and 0.5 ps are shorter than those of Ba^2+^, which is consistent with the previous study (Pappalardo et al., 2021) (Table 4). The MRTs were also calculated for other functionals, suggesting that the dispersion interaction critically affects them (Tabls S1). The lifetime of CN was also evaluated (Figure 4), which depends on $t^{*}$. The CN with the longest lifetime of CN of Ra^2+^ is eight-coordinate both for $t^{*}=0$ and 0.5 ps, and those of Ba^2+^ is seven- and eight-coordinates for $t^{*}=0$ and 0.5 ps, respectively. These results are consistent with the structural properties, i.e., the CN (Table 3) and histogram of the CNs (Figure 3). The longest lifetime of the CN of Ra^2+^ is shorter than that of Ba^2+^ for $t^{*}=0$ and 0.5 ps.

**Table 4:** Dynamic properties of Ba^2+^ and Ra^2+^

Property	$t^{*}$ (ps)	Ba^2+^	Previous study (Pappalardo et al., 2021)	Ra^2+^	Previous study (Pappalardo et al., 2021)
		This study		This study	
$N_{ex}^{t^{*}}$	0	28	–	102	–
$N_{ex}^{t^{*}}$	0.5	4	–	11	–
MRT (ps)	0	14	38	4	20
MRT (ps)	0.5	98	–	38	–

These results suggest that the water molecules in the first hydration shell of Ra^2+^ are more labile than those of Ba^2+^. Some previous results support the interpretation, e.g., Ra^2+^ has the poorest structured water molecules in the hydration shell among the alkaline earth ions (Matsuda and Mori, 2014a), and Ra^2+^ has the largest self-diffusion coefficient at infinite dilution in water among the alkaline earth ions owing to the smallest ionic potential (Yuan-Hui and Gregory, 1974).

### Comparison between radium and other alkaline earth metal ions

Barium(II) has often been used as an analog of Ra^2+^ because Ba^2+^ has an ionic radius close to Ra^2+^ and often shows similar physical and chemical properties to Ra^2+^. However, the quantitative differences between them have been scarcely discussed owing to the lack of spectroscopic and theoretical results regarding Ra^2+^. Our EXAFS and AIMD results in this study clarify the differences. As shown by the simulations and confirmed via the experiments, the CN of Ra^2+^ is larger than that of Ba^2+^. Our AIMD simulations showed that the difference in the CNs between Ra^2+^ and Ba^2+^ is owing to the difference in the dynamic motion of water molecules. Both hydrated Ra^2+^ and Ba^2+^ primarily have an eight-coordinate structure. However, the secondary structures determine the CNs of the ions: the dominant structure is nine-coordinate for Ra^2+^, while that for Ba^2+^ is seven-coordinate. In addition, structural properties, such as the tilt angle, SC, and ε, imply that the bonds between the water molecules in the first hydration shell of Ra^2+^ are weaker than those in the first hydration shell of Ba^2+^. These differences are also found in the dynamical properties: the residence time and the longest lifetime of CN of Ra^2+^ are shorter than Ba^2+^, suggesting the water molecules hydrating Ra^2+^ are more mobile than those of Ba^2+^.

From these results, it appears that Ra^2+^ is possibly more labile than Ba^2+^. Therefore, for example, the difference between Ra^2+^ and Ba^2+^ should be considered when the reaction processes between these cations and solids are considered. It has been reported that Ba^2+^ promotes the dissolution of quartz owing to the short residence time of water molecules hydrating Ba^2+^ (Dove and Nix, 1997; Dove, 1999). Our results indicate that the water molecules hydrating Ra^2+^ are more mobile than Ba^2+^ as confirmed by the shorter residence time of those hydrating Ra^2+^. Therefore, Ra^2+^ can promote dissolution to a greater extent than Ba^2+^. This chemical property can be closely related to the vitrification of radioactive waste (Gin et al., 2017) for long-term storage because radioactive wastes contain uranium and its descendant nuclides, such as Ra, and fission products (Swedish, 2011). For the removal of Ra^2+^ from groundwater, coprecipitation with barite is effective (Kraemer and Reid, 1984; Langmuir and Melchior, 1985; Beaucaire and Toulhoat, 1987; Martin et al., 2003; Vinson et al., 2013). The kinetics of coprecipitation of Ra^2+^ with barite is fast, and the activation energy is lower than Ba^2+^ and Sr^2+^ (Hedstrom et al., 2013). This behavior of Ra^2+^ can be explained by the higher mobility of water molecules hydrating Ra^2+^. The present result has an implication for incorporating Ra^2+^ into apatite (calcium phosphate), the primary step of ^223^Ra therapy for CRPC. Owing to the ionic radius of Ra^2+^ being much larger than that of Ca^2+^, the incorporation of Ra^2+^ into the bone is not favorable for its stability. However, the high mobility of water molecules hydrating Ra^2+^ shown by this study has a possibility to promote the incorporation reaction in some way, such as kinetic way.

### Conclusions

The average structure of hydration water molecules around Ra^2+^ was observed by EXAFS spectroscopy, and the values of CN and ${\bar{r}}_{Ra–O}$ were determined to be 9.2 ± 1.9 Å and 2.87 ± 0.06 Å, respectively. These values were consistent with the values of the other divalent alkaline earth ions. The results of our AIMD study provided values of 8.4 and 2.88 Å for CN and ${\bar{r}}_{Ra–O}$, respectively, and these values agree with the experimentally obtained ones. AIMD simulations were also performed to investigate the structural properties of water molecules by calculating the tilt angle between the dipole vector of a water molecule in the first shell and the vector pointing from the water oxygen to the metal ion, the SC of the distance between the first shell ion and oxygen, and the ε. The results imply that the water molecules in the first shell of Ra^2+^ are structured to a less extent than those of Ba^2+^. The dynamical properties were investigated via MRT and the lifetime of CN. The results showed shorter MRT and the shorter longest lifetime of CN for Ra^2+^ than those for Ba^2+^, suggesting the more labile hydration structure of Ra^2+^. The mobile water molecules in the first hydration shell of Ra^2+^ can explain the fast coprecipitation of Ra^2+^ with barite, and they predict that Ra^2+^ can promote the hydrolysis of quartz or other oxides to a greater extent than Ba^2+^. This study marks the beginning of a systematic scientific investigation of its transport in the environment and living organisms. These investigations contribute to improving environmental pollution owing to Ra and to the development of cancer therapy.

### Limitations of the study

A cell containing 100 H_2_O molecules and a Ra^2+^ was simulated for 60 ps by AIMD simulation in this study. This calculated cell and time can be small and short. Errors in the fitted parameters of EXAFS were estimated to be generally ±0.02 A˚ for R, ± 20% for CN, and 20% for $\sigma^{2}$ (O’Day et al., 1994).

## STAR★Methods

### Key resources table

**Table:** 

REAGENT or RESOURCE	SOURCE	IDENTIFIER
**Chemicals, peptides, and recombinant proteins**		

Sr-resin	Eichrom Technologies, Inc., USA	https://www.eichrom.com/eichrom/products/sr-resin/
Thallium oxide	FUJIFILM Wako Pure Chemical Corporation	203-00812

**Software and algorithms**		

The Vienna Ab initio Simulation Package (VASP)	Universität Wien	https://www.vasp.at/
Python version 3.9	Python Software Foundation	https://www.python.org
VESTA	Koichi Momma and Fujio Izumi	https://jp-minerals.org/vesta/en/
FEFF	University of Washington	http://monalisa.phys.washington.edu/feffproject-feff.html
REX2000	Rigaku Co., Tokyo, Japan	Version 2.5.9

**Other**		

19-element solid-state detector (SSD)	CANBERRA	GL0110S
Bag made with polyethylene and nylon	FUKUSUKE KOGYO CO., LTD.	0704997
Bag made with polypropylene	AS ONE Corporation	1-1471-01

### Resource availability

#### Lead contact

Further information and requests for resources and reagents should be directed to and will be fulfilled by the lead contact, Akiko Yamaguchi (yamaguchi.akiko@jaea.go.jp).

#### Materials availability

This study did not generate new unique reagents.

### Method details

#### EXAFS measurement

The hydration structure of Ra^2+^ was investigated at the atomic-level by EXAFS spectroscopy. The EXAFS measurements were conducted by detecting the fluorescence X-ray of Ra. The energy of Ra Lα_1_ (12.339 keV) is close to that of lead (Pb) Lβ_4_ 12.307 (keV). Thus, Pb, a descendant element of Ra, should be removed from the samples. Therefore, Ra^2+^ solution was refined with 0.001 M nitric acid using Sr-resin (Eichrom Technologies, Inc., USA) within 3 weeks before the EXAFS measurement. A solution of 50 μL containing 2 MBq Ra, corresponding to approximately 4 mM, was interfused to a sponge made by polyvinyl alcohol and packed into bags made with polyethylene and nylon thrice and a bag made with polypropylene, following the Japanese Act (Nuclear Regulation Authority, 1957) for treating radio isotope.

The EXAFS spectra were collected at BL22XU in SPring–8 (Harima, Japan). The energy of incident X-ray was monochromatized with the monochromator of two parallel Si(111) crystals and calibrated by assigning the L_I_-edge of thallium to 15.353 keV. For the calibration, a pellet of thallium oxide (Tl_2_O_3_) diluted by boron nitride was placed downstream of the ion chamber for transmission mode (I1) and upstream of the ion chamber for second transmission mode (I2). The X-ray absorption near edge structure of Tl was measured using I1 and I2 simultaneously with the Ra EXAFS measurement. The L_III_-edge EXAFS spectra of Ra were collected in fluorescence mode. The fluorescence X-ray of Ra Lα_1_ was observed by a 19-element solid-state detector (SSD) positioned at 45° to the incident X-ray. The EXAFS measurements of Ra were repeated 11 times (one scan: 60 min.) and the obtained spectra were summated.

The Ra EXAFS spectra analysis was conducted using REX2000 (Rigaku Co., Tokyo, Japan). Backscattering amplitude and phase shift were obtained by FEFF 7.0 (Ankudinov and Rehr, 1997), using a radium sulfate structure (Matyskin et al., 2017). They were plotted in Figures S1A and S1B. The curve obtained by the inverse Fourier transformation of the normalized EXAFS oscillation with a limited range from 1.84 to 2.85 Å was used for the fitting by REX2000 (Figure S1C).

#### AIMD simulation

The hydration structure of Ra^2+^ was investigated in more detail by *ab initio* molecular dynamics (AIMD) simulations based on density functional theory (DFT), using the Vienna *ab initio* simulation package (Kresse and Hafner, 1993; Kresse and Furthmüller, 1996). The dynamics of a Ra^2+^ and 100 water molecules in a cubic cell of side length 14.457 Å with periodic boundary conditions were calculated using Γ-only k-point DFT. For the calculation of the hydrated Ra^2+^ system, the total valence state of the cell was adjusted to +2, and a jellium background was introduced for charge neutralization. The Nosé–Hoover thermostat was used for the NVT simulation with a time step of 0.25 fs at 330 K (Nosé, 1984; Hoover, 1985). This temperature is suitable for the simulation of bulk water using the strongly constrained and appropriately normed (SCAN) meta-GGA exchange-correlation (XC) functional (Chen et al., 2017; Zheng et al., 2018; LaCount and Gygi, 2019; Xu et al., 2019; Dellostritto et al., 2020; Duignan et al., 2020a, 2020b).

This simulation in the main text was conducted with the SCAN meta-GGA XC functional (Sun et al., 2015). In general, the results of DFT calculations depend on the XC functionals. Therefore, the AIMD simulations with the Perdew–Burke–Ernzerhof (PBE) (Perdew et al., 1996), Becke–Lee–Yang–Parr (BLYP) (Becke, 1988; Lee et al., 2011), PBE-D3, and BLYP-D3 (Grimme, 2006; Grimme et al., 2010; Grimme et al., 2011) GGA XC functionals were performed for comparison. The PBE-D3 and BLYP-D3 GGA XC functionals include the zero damping DFT-D3 dispersion interaction correction (Grimme et al., 2010). For all calculations, snaps from 10 to 60 ps were analyzed after 10 ps equilibration. The time evolutions of the total energies of the hydrated Ra^2+^ and Ba^2+^ systems with these XC functionals were evaluated to confirm their thermal equilibriums (Figure S2).

### Quantification and statistical analysis

EXAFS fitting was performed using FEFF 7.0 (Ankudinov and Rehr, 1997) and REX2000 (Rigaku Co., Tokyo, Japan). Analysis of AIMD simulation was conducted based on Equations 1, 2, and 3 described in the main text.

## Data Availability

• Data reported in this paper will be shared by the lead contact upon request. • This paper does not report original codes. • Any additional information required to reanalyze the data reported in this paper is available from the lead contact upon request.
