# Supplementary material for: Extended X-ray absorption fine structure spectroscopy measurements and ab initio molecular dynamics simulations reveal the hydration structure of the radium(II) ion
Source: iScience. 2022 Jul 19;25(8):104763. doi: 10.1016/j.isci.2022.104763 (PMC9386089; doi:10.1016/j.isci.2022.104763)
Supplement: Document S1. Figures S1–S8 and Table S1 [file mmc1.pdf]

iScience, Volume *25*

Supplemental information

Extended X-ray absorption fine structure spectroscopy measurements and *ab initio* molecular dynamics simulations reveal the hydration structure of the radium(II) ion

Akiko Yamaguchi, Kojiro Nagata, Keita Kobayashi, Kazuya Tanaka, Tohru Kobayashi, Hajime Tanida, Kojiro Shimojo, Tetsuhiro Sekiguchi, Yui Kaneta, Shohei Matsuda, Keiichi Yokoyama, Tsuyoshi Yaita, Takashi Yoshimura, Masahiko Okumura, and Yoshio Takahashi

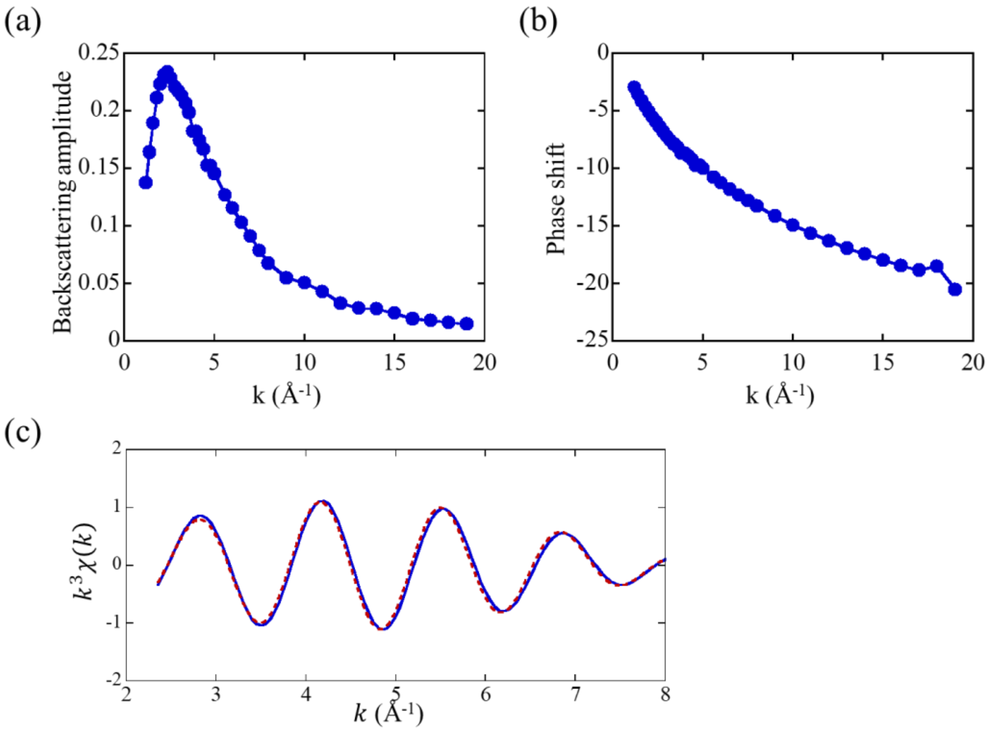

Fig. S1. Constants used in EXAFS analysis and analyzed EXAFS spectra, related to STAR Methods. (a) Backscattering amplitude and (b) phase shift constants. (c) The Fourier transformation of the normalized EXAFS oscillation (blue solid line) and fitted curve (broken red line).

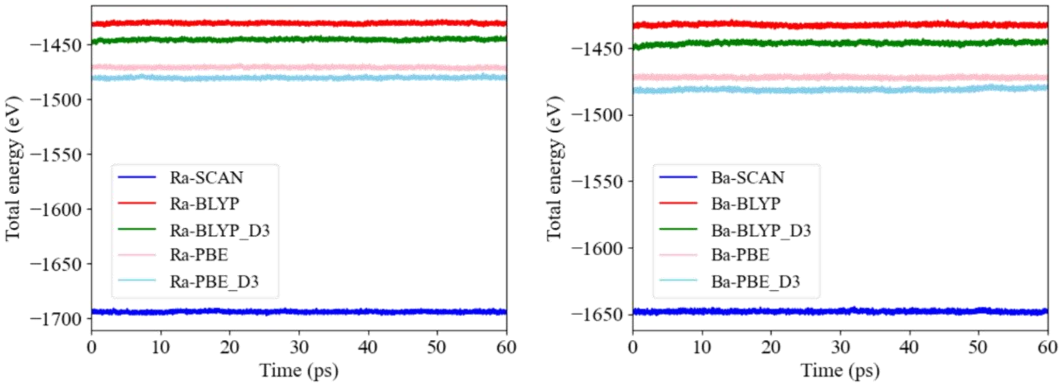

Fig. S2. Time evolution of the total energies of the hydrated Ra^{2+} and Ba^{2+} systems with the SCAN, BLYP, BLYP-D3, PBE, and PBE-D3 XC functionals, related to STAR Methods. The horizontal and vertical axes represent simulation time and total energy of the system, respectively. The blue, red, green, pink, and light blue lines represent the total energies with the SCAN, BLYP, BLYP-D3, PBE, and PBE-D3 XC functionals, respectively.

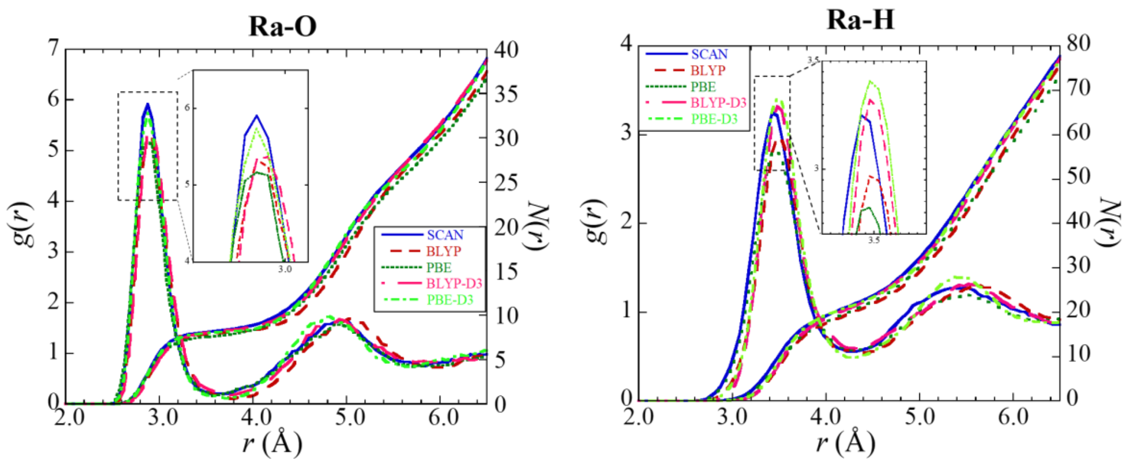

Fig. S3. Radial distribution functions $g(r)$ and running integration numbers $N(r)$ of (a) Ra^{2+}–O and (b) Ra^{2+}–H, related to Fig. 2.

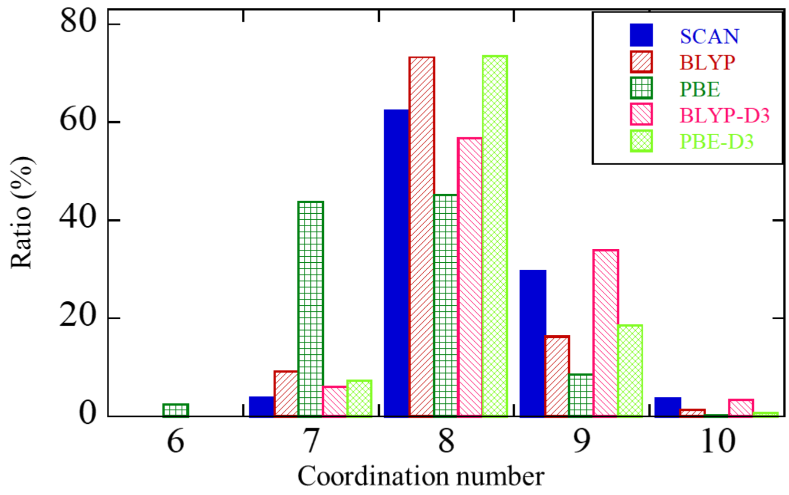

Fig. S4. Histograms of the oxygen atoms in the water molecules around Ra^{2+}, related to Fig. 3. The blue, red diagonal lined, green lattice, magenta diagonal lined, and light green diagonal lattice bars represent the frequency evaluated by the SCAN, BLYP, PBE, BLYP-D3, and PBE-D3 XC functionals, respectively.

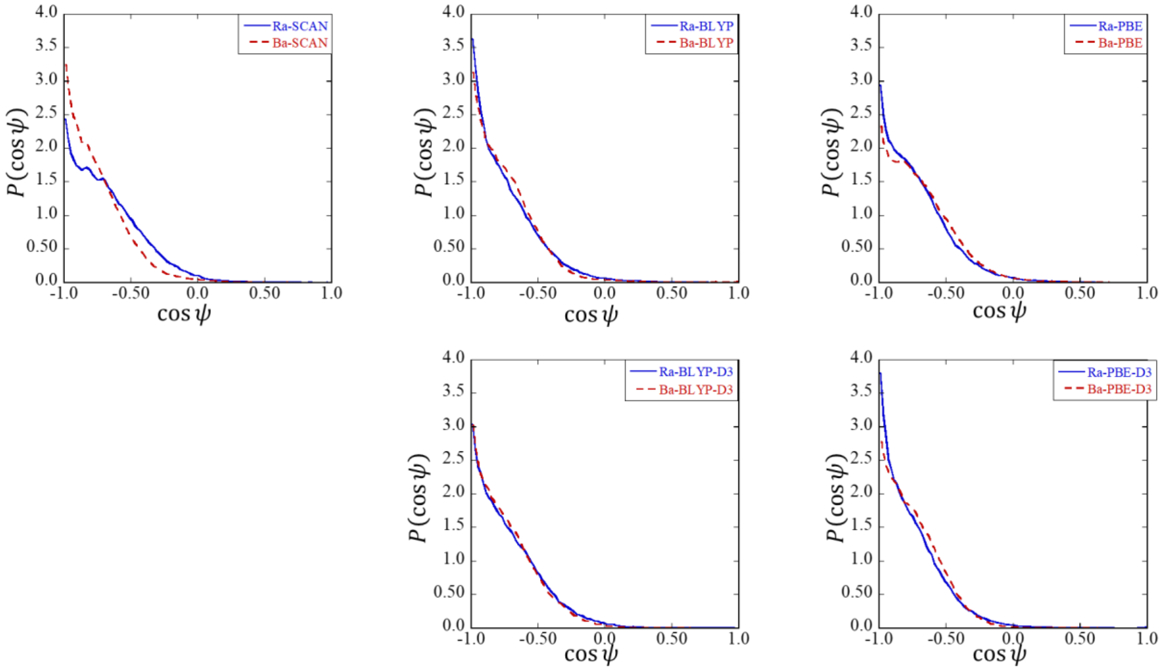

Fig. S5. Probability distribution of $\cos\Psi$ obtained by the DFT calculations with the SCAN, BLYP, BLYP-D3, PBE, and PBE-D3 XC functionals, related to Fig. 3. The blue line and red dashed line represent the probability distributions of $\cos\Psi$ for Ra^{2+} and Ba^{2+}, respectively.

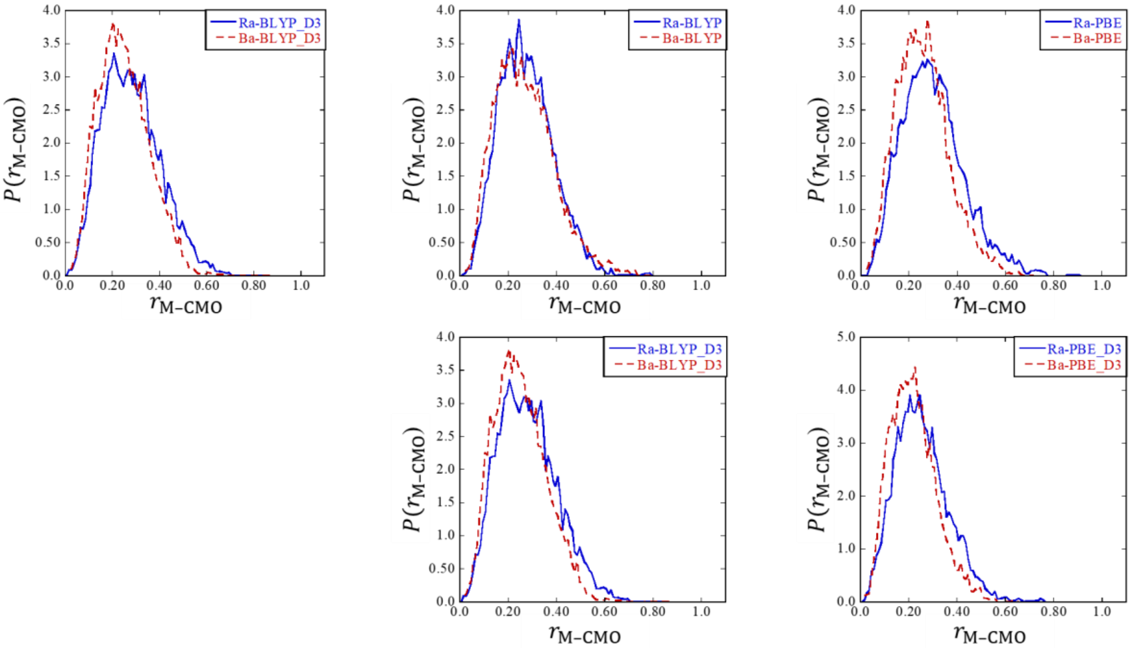

Fig. S6. Probability distribution of $r_{\text{M-CMO}}$ obtained by the DFT calculations with the SCAN, BLYP, BLYP-D3, PBE, and PBE-D3 XC functionals, related to Table 3. The blue line and red dashed line represent the probability distributions of $r_{\text{M-CMO}}$ for Ra^{2+} and Ba^{2+}, respectively.

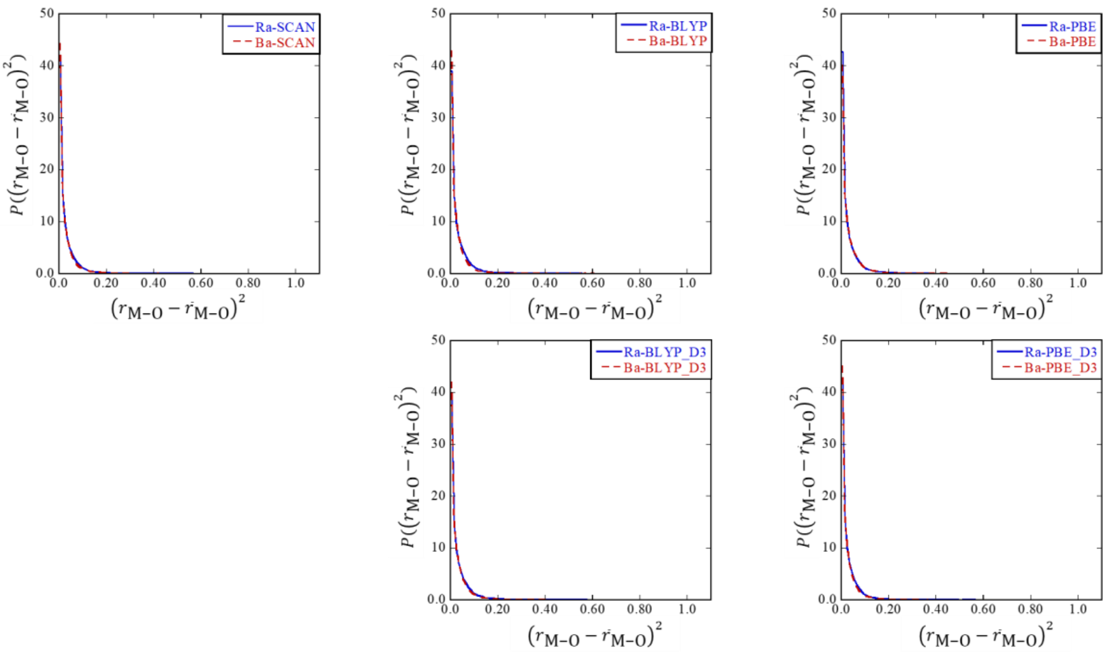

Fig. S7. Probability distributions of $(r_{\text{M-O}} - \bar{r}_{\text{M-O}})^2$ obtained by the DFT calculations with the SCAN, BLYP, BLYP-D3, PBE, and PBE-D3 XC functionals, related to Table 3. The blue line and red dashed line represent the probability distributions of $(r_{\text{M-O}} - \bar{r}_{\text{M-O}})^2$ for Ra^{2+} and Ba^{2+}, respectively.

Table S1. The MRT and $N_{ex}^{t^*}$ values for Ba^{2+} and Ra^{2+} evaluated by the AIMD simulations with the SCAN, BLYP, BLYP-D3, PBE, and PBE-D3 XC functionals, related to Table 4. The classical MD results in the previous study are listed for comparison.

t^* (ps)	XC-functional	Ba^{2+}		Ra^{2+}	
		$N_{ex}^{t^*}$	MRT (ps)	$N_{ex}^{t^*}$	MRT (ps)
0 ps	SCAN	28	14	102	4
	BLYP	50	7	52	8
	BLYP-D3	71	6	85	5
	PBE	72	6	80	5
	PBE-D3	28	14	58	7
	Classical MD (Pappalardo *et al.*, 2021)	–	38	–	20
0.5 ps	SCAN	4	98	11	38
	BLYP	12	30	12	34
	BLYP-D3	17	24	21	20
	PBE	16	25	17	22
	PBE-D3	4	98	5	81
2 ps	SCAN	3	130	3	140
	BLYP	5	73	4	101
	BLYP-D3	8	52	12	35
	PBE	4	100	10	38
	PBE-D3	2	195	3	135
	Classical MD (Pappalardo *et al.*, 2021)	–	53	–	29

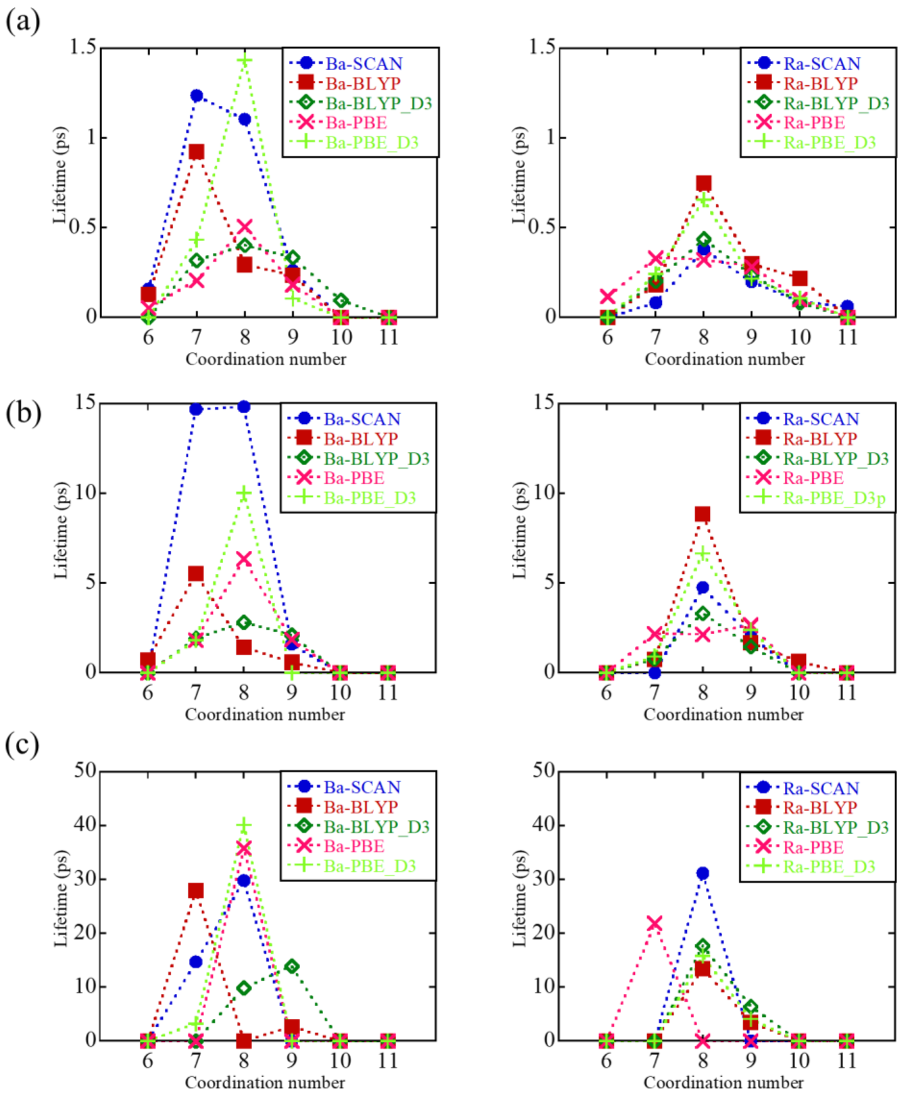

Fig. S8. The lifetime of CN for (a) $t^* = 0$ ps, (b) $t^* = 0.5$ ps, and (c) $t^* = 2$ ps, related to Fig. 4. The blue circle, red square, green diamond, pink cross, and light green plus represent the results with the SCAN, BLYP, BLYP-D3, PBE, and PBE-D3 XC functionals.
